# Implementation of High and Intensive Care (HIC) in the Netherlands: a Process Evaluation

**DOI:** 10.1007/s11126-021-09906-x

**Published:** 2021-03-26

**Authors:** A. Laura van Melle, Alida J. van der Ham, Guy A. M. Widdershoven, Yolande Voskes

**Affiliations:** 1 Department of Ethics, Law and Humanities, Amsterdam UMC, Amsterdam, Netherlands; 2 GGZ inGeest, Amsterdam, The Netherlands; 3 Mental Healthcare Centre GGZ Breburg, Tilburg, The Netherlands; 4 Tilburg School of Social and Behavioral Sciences, Tranzo Scientific Center for Care and Welfare, Tilburg University, Tilburg, The Netherlands

**Keywords:** High and intensive care, Inpatient psychiatry, Implementation, Coercion, Quality of care

## Abstract

The High and Intensive Care model (HIC) was developed to reduce coercion and improve the quality of acute mental health care in the Netherlands. This study aimed to identify drivers of change which motivate professionals and management to implement HIC, and to identify facilitators and barriers to the implementation process. 41 interviews were conducted with multiple disciplines on 29 closed acute admission wards for adult psychiatric patients of 21 mental healthcare institutions in the Netherlands. The interviews were analysed by means of thematic analysis, consisting of the steps of open coding, axial coding and selective coding. Findings reveal three major drivers of change: the combination of existing interventions in one overall approach to reduce coercion, the focus on contact and cooperation and the alignment with recovery oriented care. Facilitators to implementation of HIC were leadership, involving staff, making choices about what to implement first, using positive feedback and celebrating successes, training and reflection, and providing operationalizable goals. Barriers included the lack of formal organizational support, resistance to change, shortage of staff and use of flex workers, time restraints and costs, lack of knowledge, lack of facilities, and envisaged shortcomings of the HIC standards. Drivers of change motivate staff to implement HIC. In the process of implementation, attention to facilitators and barriers on the level of culture, structure and practice is needed.

## Background

Since the beginning of this century, the prevention and reduction of coercion in psychiatry has been a topic for debate in the Netherlands. In 2006, the Dutch branch organization for mental healthcare (GGZ Nederland) formulated the aim to reduce seclusion and other coercive measures by 10 % yearly. Supported by the Dutch government, several projects have been started in the Netherlands since 2006 to reduce coercion, and mainly seclusion [2]. As a result of the development of many interventions within these projects, considerable reduction of seclusions was achieved, albeit not as large as was aimed for. Moreover, some mental healthcare institutions did achieve a reduction in line with the aims, while others did not. One of the explanations is that the reduction of coercion is not only a matter of developing new interventions but requires a change in organizational structure, culture and practices, including stable and motivated management and support at all levels of the organization [22].

A new national approach for acute mental healthcare was needed to further reduce coercion and to simultaneously strive for better quality of care. In 2013, the HIC model was developed, which focusses on restoring and maintaining contact, crisis prevention and stepped care [27]. The HIC model and its development are described in detail elsewhere [26, 29]. Implementation of new care approaches such as HIC is not straightforward process. Previous studies have shown that implementation of interventions aimed at reducing coercion may require changes in leadership, training and education of staff, monitoring seclusion rates and a change of the ward environment [7, 11, 25, 28]. More insight is needed to identify elements that influence the implementation of the new HIC model.

What drives professionals to take up HIC? Insight into the experienced drivers of change can explain the motivation to embrace the HIC model. Drivers of change may function as a catalyst by creating momentum and expressing a sense of urgency combined with a clear vision [8, 16]. Also, facilitators and barriers to the implementation process of HIC are relevant. Literature on implementation suggests that a careful analysis of facilitators and barriers to the implementation process can help to make timely adjustments to the implementation process and aid to secure interventions into policy [10, 12, 15]. Therefore, the aim of this study is to identify the drivers of change, as well as facilitators and barriers in the implementation process of HIC.

## Methods

### Design

Qualitative research was conducted using semi-structured individual and group interviews with mental health professionals.

### Wards and Participants

The interviews were conducted on 29 closed acute admission wards for adult psychiatric patients (18 years and older) of 21 mental healthcare institutions in the Netherlands that participated in the development and implementation of HIC. The institutions differed in the stage of implementation of HIC. The managers of participating institutions were asked to recruit staff who had experience with the HIC model and were involved in the implementation process at the ward. Participants consisted of staff working at the wards, and were selected by means of purposive sampling [4] to achieve maximum diversity regarding disciplines [18]. Most participants were nurses/ nurse specialists (*n* = 28), psychiatrists/ psychologists (*n* = 9) and managers/ directors (*n* = 7). Also other disciplines were included; a social worker (*n* = 1), a psycho-motoric therapists (*n* = 1), a peer provider (*n* = 1), a nursing scientist (*n* = 1), and a quality officer (*n* = 1). In total, 49 participants were interviewed.

### Data Collection

The data were collected between February 2014 and May 2015. A total of 41 interviews were held, of which 33 individual interviews and eight group interviews. The interviews were carried out by the first author (LvM). A topic list was used. Questions asked were for example “How do you experience working according to the HIC approach?”, “What added value does HIC have in your perspective?”, “How do you think the implementation of HIC is progressing at the ward?”, “Which elements contribute to a better implementation?”, and “Which barriers do you encounter in the implementation process of HIC?”. Interviews lasted for approximately 1 h and took place at the participant’s work location, usually on the HIC ward. The interviews were recorded and transcribed verbatim.

### Ethical Considerations

All participants received written information on the purpose of the study and were asked to sign an informed consent form prior to the interview. The Medical Ethics Review Committee of the Amsterdam University Medical Center declared that the study did not require specific ethics approval. This study was approved by the Amsterdam Public Health Research Institute.

### Analysis

The analysis of the data was based on an iterative process, meaning that the analysis of the data started during the period of data collection [23]*.* The interviews were analysed by means of thematic analysis. The first step consisted of the steps of open coding of transcripts. Next, data were analysed deductively by means of axial coding and selective coding [5], based on the following three predetermined categories: drivers of change, facilitators and barriers. For each category, underlying themes were identified. To increase the validity of the study, the interviews were interpreted by multiple researchers (LvM, YV & GW) [4, 6]. Reports of the interviews were provided to the participants as a member check to ensure the correctness of the interpretation of the interviews by the researchers [19, 24].

## Results

This section will first discuss the results with regard to the drivers of change. Second, the facilitating factors and the barriers to the implementation process will be presented.

### Drivers of Change

We found three drivers of change: 1) HIC combines existing interventions in one overall approach to reduce coercion; 2) HIC focuses on contact and cooperation; and 3) HIC is in line with recovery oriented care.

- *HIC combines existing interventions in one overall approach to reduce coercion*

Many participants reported to see the HIC model as a culmination of efforts to reduce coercion in the past. They mentioned that HIC takes these efforts a step further by bringing attention to the urgency to keep working on the reduction of coercion, clearly linking these efforts to quality of care and by providing concrete guidelines for professionals.

“We were already working on reducing coercion…but in mental health care we need a ‘trigger’. HIC offers an incentive to keep improving and to take action in reducing coercion” (psychiatrist)

“We have already taken many steps to reduce coercion, but the ward environment still needs many changes. HIC makes it concrete….HIC has been a way to ensure continued development…” (manager)

- *HIC provides a focus on contact and cooperation*

Participants stressed the need to focus on contact with patients to facilitate recovery. The HIC model offers them a framework for restoring and maintaining contact with patients, and thereby provides an alternative to routines and rules that were based on control. A team leader said:

“We had to transform a ward that was good at controlling, almost in a forensic way, while we just have regular psychiatric patients, no forensic patients, but still seven pages with ward rules. This had to change.”

A nurse describes the need for better collaboration with outpatient services to ensure continuity of care. The HIC approach fosters a better alliance with care partners such as outpatient teams:

“I notice that the collaboration with outpatient teams has improved, which is very important, we know how to find each other better…. Now it’s better coordinated... I think this was already something that was needed, but I don't know if we would have started working on this without HIC.” (nurse)

- *HIC is in line with recovery oriented care*

The HIC model aligns with a recent development in mental health care, namely the focus on recovery oriented care. By emphasizing self-determination, connectedness and self-management HIC incorporates some of the core elements of recovery oriented care. A manager said:

“It is a great guideline to transform a ward in a short period of time into something that also completely fits within the philosophy of the institution at the moment. ‘Recovery is feasible’, ‘recovery takes place at home’; these concepts are highly relevant!”

The HIC model also contributes to creating a “healing” ward environment with concrete standards for high quality care focused on recovery. A psychiatrist explained:

“With the HIC approach I want to work towards a ward where the care is so good, I would in a matter of speaking, be willing to admit my sister to.”

### Facilitators and Barriers

Although HIC inspires to change practice, conditions are needed to actually realize HIC and barriers must be overcome. This section describes the facilitators and barriers in the implementation of HIC that were experienced by participants.

### Facilitators

#### Leadership

Participants emphasized the importance of a clear management style and good communication with the team about the changes needed. A manager mentioned the need to set norms for implementation of HIC:

“We do not allow a discussion on whether or not we are going to do this [implementing HIC]. We present the HIC approach as a norm. Than they just have to make it their own and act accordingly.” (manager)

Nurses emphasized that support from management to innovate and to strive for a further reduction of seclusion is necessary, as it increases staff motivation to take more risks and to be more creative. Also a director agreed with this:

“You have to support it if someone dares to do that [to go outside with a patient], even if that patient may run away. So we should not reprimand someone whenever they think out of the box.”

#### Involving Staff

Next to top-down initiatives and support, a bottom-up approach is also needed. Participants indicated that it is important to organize team meetings, to create project or working groups and to discuss ideas regarding implementation strategies. This can assist in setting goals, taking responsibility for these goals and evaluating the outcome. HIC should not feel as another set of rules, but something that nurses want to embrace to improve quality of care. A manager said:

“The project group consists of employees who are present on the work floor. So the input comes from practice… That works because you give them [HIC staff] responsibility.”

#### Making Choices about What to Implement First

According to participants, implementation of the HIC model requires making choices and prioritizing. A step-by-step approach, in which one should try to avoid implementing too many interventions at the same time, is needed. A nursing scientist explained:

“The risk is that not everyone keeps up with the developments, and that you get incomplete developments. It is better to implement at a slower rate than doing it all at once and risking that nobody knows what they are doing.”

Before planning next steps it is important to evaluate what went well and what needs improvement. A careful planning process was considered to increase its success and therefore create feelings of achievement. A nurse said:

“We have to take the time to do it right and together, so that the chances of success and benefits are well secured. When it is done too quickly, it might explode because you have not properly secured it.”

#### Using Positive Feedback and Celebrating Success

Positive feedback on improvements made in relation to HIC is experienced as highly motivating. A nurse said:

“Many patients who were previously admitted at the old ward said they absolutely dreaded to be admitted here again. However, later on they told us they really appreciated the new way of working, especially the hospitable treatment, welcoming attitude, and less controlling behaviour.”

Moreover, it is important to share success stories with each other and also to share the feeling of pride. This stimulates the willingness to change. It is also important to celebrate successes. A nurse indicated:

“A success story makes you want to try again, because you noticed it worked! It requires effort, but it is something that empowers the team. We can do this together. It works miracles.”

#### Training and Reflection

Education for nurses and training of competences were seen as essential to successful implementation of the HIC approach. Training provides staff with the required knowhow and with the confidence to provide intensive care to their patients. Through training participants learn how to apply principles of HIC in their daily work. A nurse explained:

“Training in conversation techniques for different crisis situations can make escalation less likely. The application of these techniques that suit the situation can increase security and improve hospitality on the ward.” (nurse)

Several participants stressed the importance of reflection and feedback in order to keep improving quality of care at the ward:

“Openness to feedback is very important and others should be asked for advice. The ward should stay in development.” (psychiatrist)

#### Providing Operationalizable Goals

Participants stated that the HIC workbook provides a clear vision and concrete working methods. The HIC monitor is said to provide guidelines to improving care. Also audit results on the HIC monitor were mentioned as a positive factor.

“By participating in the audits we hope to address streamlining the care within our teams and improve collaboration. The audits also offer the opportunity to look how other wards are doing and the exchange of experiences is inspiring to us.” (manager)

Managers also valued creation of a learning network of auditors in which experiences and knowledge were exchanged. Participants also valued the audits to help reflect on the implementation process. A nurse said:

“Our intention is to evaluate regularly, but we’re quickly swayed by the issues of the day. That is why I value the audit to stop to reflect and to get a clear overview of how things are going…it helps us to safeguard interventions into working practices.”

### Barriers

#### Lack of Formal Organizational Support

Firstly, the lack of a formal fiat from the organization to start implementing HIC was often mentioned to be a barrier. This meant that less resources were made available by the organization and staff felt less inclined to adopt a new vision when this vision was not officially supported by the organization. A nurse explained that the direction of the organization was unclear to many, which made further planning of implementation difficult:

“I did receive the HIC workbook but I didn’t read it because it is still unclear when and where we will start with HIC. At this point we do not know which direction to go and therefore we cannot focus on for example training and planning an implementation strategy.”

#### Resistance to Change

Difficulties during the implementation process of HIC also included staff members showing resistance. A team leader stressed that not everyone within the team is able to adjust to the new ward culture and ways of working:

“A culture has been built up here for six, seven years and you don't change in a year. That just takes time. This sometimes also costs people who work here, who then no longer feel comfortable with the new way of working.”

A manager said that having a psychiatrist on the team who does not want to change had a negative impact on the rest of the team and possibilities to innovate. Resistance to change was also present in the collaboration with the outpatient care. Nurses, psychiatrists and managers indicated that many outpatient care workers are reluctant to being involved with the care process in the clinic, and to frequently visit the ward. At some wards this resulted in longer admissions. A psychiatrist commented:

“I noticed we are not yet aligned in vision. The HIC approach states when the crisis is over, a patient should go home. However, outpatient care might indicate to wait and let the patient stay a little longer at the clinic without it really substantively contributing to the healing process.”

#### Shortage of Staff and Flex Workers

Many wards experience difficulty to fill available positions for different disciplines such as nurses, psychiatrists, nurse practitioners and peer experts. Staff members frequently indicate that their current staff size is too small to always be able to provide one-to-one care. These shortages, vacancies and an increased use of flex personnel and can have negative consequences for team cohesion and continuity of care. As many flex workers are unfamiliar with the HIC approach, this can stagnate innovation and lead to an increase of coercive measures. A team leader explained:

“If you look at how the HIC is officially classified in terms of staffing and you really want to work according to HIC principles, then you really need more personnel. We can't live up to that now and on that part implementation is stagnating.”

Nurses reported that the shortage of staff and flex workers also have a negative effect on feelings of safety. The ability to trust co-workers is necessary to provide one-to-one care in situations where patients would previously have been secluded. Feeling unsafe impedes innovation. A nurse said:

“Whenever you feel less safe, you tend to adhere to the rules, It cannot be done, it cannot succeed, It doesn’t work. Whenever you do feel safe, you are willing to try.”

#### Changes Take Time and Costs Are High

According to participants, it takes time to bring about the wanted changes needed for implementation of HIC. For example, drafting plans for renovations and the realization of these plans to build the intensive care units take time. Often, these plans cause discussion within the team and organization, which can further delay the start. Moreover, disagreement about investments can further distance management from nursing staff. Some interviewed managers wanted to prioritize investment in facilities, whilst several nurses from these locations would rather see an increase in number of staff. A peer provider said:

“You can build astonishing HIC facilities, but if you have a terrible staff you will get nowhere. I think a good team is much more valuable than an adjusted building.”

Financial barriers include high staffing costs, which makes many managers concerned about the feasibility of adhering to the standards to achieve full implementation of the HIC approach. A manager expressed his concern that these standards of care will not be cost-effective. This proves to be of extra difficulty for smaller wards, who will have to meet the same standards but have less revenue. A team leader mentioned:

“It is a big [financial] drain if you want to organize hand-in-hand care, seven days a week, 24 hours a day. And if you don't have enough [staff] formation, how will you do it? Especially at night and on weekends. We are currently struggling with that.”

#### Lack of Knowledge

Nurses mentioned that they experienced a lack of knowledge about specific interventions, such as how to best provide one-on-one care. Also, having to work with new instruments that can be experienced as substituting nurses’ own competence and intuition can foster feelings of insecurity. An example is mentioned by a nurse concerning uncertainties with rooming-in of relatives at the ward, while dealing with privacy and safety concerns. A nurse commented on the provision of one-on-one care:

“It is not clear for everyone what intensive care exactly is, the definitions are not always clear (...) One-on-one care, how do you practice that? For which patients is it appropriate and for which is it not? It is a continuing quest.” (nurse)

#### Lack of Facilities

Difficulties are encountered due to technical limitations and the current building structure which do not always support HIC working methods. For example, nurses report some patient rooms to be too small to provide good one-on-one care, to not have their own bathroom, or a bathroom with only cold water. The IC is often seen as an essential condition to avoid seclusion, and without it no other solution to reduce coercion is seen:

“There are patients who are too restless or too aggressive to be at the ward and for whom seclusion is just a bit too much, and not necessary. We now have a patient who we would rather have on an IC, but because we don’t have it yet and other facilities are still missing, he is now secluded. If we would have more staff or space we could just take him out of the seclusion room.” (nurse)

#### Envisaged Shortcomings of the HIC Standards

A final barrier in the implementation experienced by respondents refers to the HIC standards. Some stakeholders criticized elements of the HIC model. Notably, the idea of a High Security Room (HSR) was seen as contradictory to the philosophy of HIC to not seclude patients:

“In my view, the HSR (EBK) is just a disguised seclusion room…We don't want to lock people up anymore, but we still invest in such a space.” (team leader)

Also, several participants missed elements in the model, such as guidelines on how to provide one-on-one care and handling emergency scenarios and detailed descriptions of competences and tasks of nurses, psychologists, nurse practitioners and peer experts.

## Discussion

This paper analyses the most important drivers of change to embrace the HIC model, and the facilitators and barriers characterizing the implementation process in 21 mental healthcare institutions in the Netherlands. Findings reveal three major drivers of change: the combination of former initiatives in an overall approach to reduce coercion, the alignment with recovery oriented care and the focus on contact and cooperation. These drivers of change explain the motivation to start the implementation of the HIC model. In the earlier coercion reduction projects the focus was generally on separate elements. Moreover, these were framed in a negative way, emphasizing what should be prevented or reduced - seclusion in particular – while attention for positive motivation to change was limited. The drivers of change we identified in our study show the strength of a positive framing of new working methods.

Although the drivers of change provide motivation to implement HIC, actually adopting new working routines requires changing deeply rooted structures and culture at wards and institutions [17]. Attention for barriers and facilitators may help to steer implementation process in the desired direction. We will elaborate on the barriers and facilitators that were identified in this study and discuss them in terms of culture, structure and practice.

In our study, barriers related to *culture* were most evident in the resistance to change among some professionals. These findings highlight that changing culture takes time and that additional efforts are needed to address resistance among key stakeholders such as psychiatrists and outpatient care professionals. Our study suggests that actively involving staff in setting goals related to HIC may help to realize a change in culture. Previous studies showed that active involvement of staff in an early stage of an implementation process helps to address their needs and diminishes resistance to change [13, 14, 21]. Also, leadership supporting HIC was identified in this study as an element which could facilitate a change in culture. This is in line with the description of good leadership in literature, which is said to change staff’s willingness to change and beliefs about the new HIC model and its effectiveness [3, 13]. Previous research on reduction of coercion found psychiatrists to be influential; when a psychiatrist shows commitment to the transition towards HIC, this will facilitate the shared vision, joint responsibility and trust [1]. Other strategies identified in this study and that may be helpful in realizing a culture change include the use of positive feedback and celebration of successes.

Several barriers identified in this study can be related to *structure*, including a shortage of staff and facilities, the lack of a formal institutional policy and the costs associated with implementation of HIC. A lack of resources is a common barrier in implementation of services in mental healthcare [10, 20]. Potential facilitators that emerged in our study include having an implementation plan to prioritize and structure the different aspects of the implementation process at an organizational level. Also, insight into the potential (cost)effectiveness of the intended change may be beneficial in allocating means for the implementation process. The HIC workbook and the monitor provide structural elements needed for the implementation of HIC at the workplace level [26].

This study showed that implementation of new *practices* requires a planning process in which a limited number of changes are promoted. This may help to address the barrier of time pressure, which causes people to stick to old routines [9]. The HIC workbook and the monitor provide concrete descriptions of the envisioned working practices according to the HIC model and can facilitate the incorporation of these practices in daily routines of professionals at wards. In addition, providing training about HIC interventions may support professionals to avoid coercion and take a pro-active approach in their daily work [13]. Our study also indicates that stimulating reflection on care practices and quality of care helps professionals to evaluate current habits and routines and consider new ways of working.

The implementation of HIC focused on change in culture and practice. Facilitators in these domains were perceived to be effective, and barriers were often addressed. On the level of structure, barriers are more persistent. Shortage of employees and lack of funding and facilities are driven by organizational and national policies, the labor market and the need to be cost-effective in providing care. As such, implementation of HIC may not always be prioritized in budgets and organizational plans, while for HIC to be successful budget and resources need to be allocated.

### Strengths and Limitations

A strength of this study is the nationwide scope, assessing experiences with and views on the implementation of HIC among a diverse group of managers and staff of HIC wards. Another strength is that the interviews were held in the first two years of the implementation of HIC, while wards differed in stages of implementation. This provided in-depth insights into the drivers of change, barriers and facilitators in various wards.

A limitation of this study is a potential bias in the selection of respondents. Many participants were actively involved in project groups set up to implement HIC, and may have been inclined to be supportive of the HIC model compared to professionals not involved in such groups. Another limitation is the limited generalizability beyond the context of acute mental healthcare in The Netherlands. Findings from this study however correspond with outcomes of studies in different contexts and countries [9, 30]. This suggests that the results could potentially be relevant for other settings, and especially other mental healthcare setting such as long term mental healthcare and outpatient mental healthcare, for which setting-specific drivers, barriers and facilitators need to be taken into account.

## Conclusion

The HIC model is fostered by three drivers of change: the combination of existing interventions to reduce coercion in a systematic way, the focus on contact and cooperation, and the alignment with recovery oriented care. The implementation of HIC is facilitated by leadership, involving staff, prioritizing goals and activities, using positive feedback and celebrating successes, and providing operationalizable goals in the HIC workbook, monitor and audits. Barriers included the lack of formal organizational support, resistance to change, shortage of staff and use of flex workers, time restraints and costs, lack of knowledge, lack of facilities, and envisaged shortcomings of HIC standards. Improving the complex system of care in acute admission wards requires positive motivation through drivers of change, as well as attention for facilitators and barriers on the level of culture, structure and practice.
